# 3-[(3-Oxo-1,3-dihydro­isobenzofuran-1-yl)amino]benzoic acid

**DOI:** 10.1107/S1600536809038926

**Published:** 2009-09-30

**Authors:** Wenkuan Li, Handong Yin, Liyuan Wen, Kang Li, Weidong Fan

**Affiliations:** a College of Chemistry and Chemical Engineering, Liaocheng University, Shandong 252059, People’s Republic of China

## Abstract

In the title compound, C_15_H_11_NO_4_, the dihedral angle formed by the benzene ring and isobenzofuran ring system is 67.82 (5) Å. The crystal structure is stabilized by inter­molecular O—H⋯O and N—H⋯O hydrogen-bonding inter­actions.

## Related literature

For general background to isobenzofuran derivatives, see: Landge *et al.* (2008 ▶); Paradkar *et al.* (1998 ▶); Joseph (1998 ▶). Odabaşoğlu & Büyükgüngör (2008 ▶).
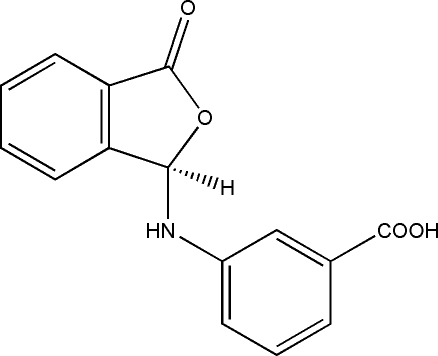


## Experimental

### 

#### Crystal data

C_15_H_11_NO_4_
*M* *_r_* = 269.25
Monoclinic, 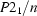

*a* = 10.9025 (15) Å
*b* = 8.1595 (12) Å
*c* = 14.2654 (18) Å
β = 103.463 (1)°
*V* = 1234.2 (3) Å^3^
*Z* = 4
Mo *K*α radiation
μ = 0.11 mm^−1^
*T* = 298 K
0.27 × 0.19 × 0.17 mm

#### Data collection

Siemens SMART CCD area-detector diffractometer
Absorption correction: multi-scan (*SADABS*; Sheldrick, 1996 ▶) *T*_min_ = 0.972, *T*_max_ = 0.982
6011 measured reflections
2171 independent reflections
1206 reflections with *I* > 2σ(*I*)
*R*_int_ = 0.038

#### Refinement

*R*[*F* ^2^ > 2σ(*F* ^2^)] = 0.041
*wR*(*F* ^2^) = 0.107
*S* = 0.90
2171 reflections
181 parameters
H-atom parameters constrained
Δρ_max_ = 0.23 e Å^−3^
Δρ_min_ = −0.13 e Å^−3^

### 

Data collection: *SMART* (Siemens, 1996 ▶); cell refinement: *SAINT* (Siemens, 1996 ▶); data reduction: *SAINT*; program(s) used to solve structure: *SHELXS97* (Sheldrick, 2008 ▶); program(s) used to refine structure: *SHELXL97* (Sheldrick, 2008 ▶); molecular graphics: *SHELXTL* (Sheldrick, 2008 ▶); software used to prepare material for publication: *SHELXTL*.

## Supplementary Material

Crystal structure: contains datablocks I, global. DOI: 10.1107/S1600536809038926/bq2162sup1.cif

Structure factors: contains datablocks I. DOI: 10.1107/S1600536809038926/bq2162Isup2.hkl

Additional supplementary materials:  crystallographic information; 3D view; checkCIF report

## Figures and Tables

**Table 1:** Hydrogen-bond geometry (Å, °)

*D*—H⋯*A*	*D*—H	H⋯*A*	*D*⋯*A*	*D*—H⋯*A*
O1—H1*A*⋯O4^i^	0.82	1.91	2.712 (2)	166
N1—H1⋯O2^ii^	0.86	2.16	2.956 (2)	154

Symmetry codes: (i) 
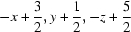
; (ii) 
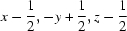
.

**Fig. 1.**
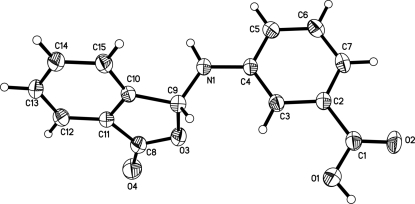
The asymmetric unit of (I), showing 50% probability displacement ellipsoids.

**Fig. 2.**
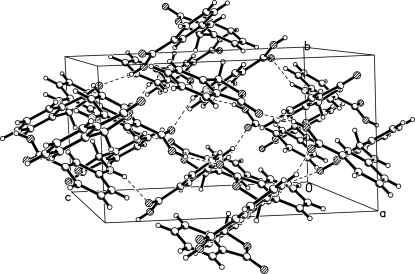
Crystal packing of (I) with hydrogen bonding as dashed lines.

## supplementary crystallographic information

### Comment

Phthalides (isobenzofuran-1(3*H*)-ones) are well known for their interesting biological properties (Paradkar *et al.*, 1998; Joseph, 1998). In addition, 3-substituted phthalides are vital heterocyclic motifs in many bioactive compounds such as isocoumarins, anthraquinones, anthracyclines, and several alkaloids (Landge *et al.*, 2008). In view of this, various methods have been reported for their synthesis. Herein, the crystal structure of the title compound is presented.

The title compound, (I), (Fig. 1) is a chirality compound with a chiral center at C_9_. The dihedral angle between the benzene ring and isobenzofuran ring system is 67.82 (5) Å indicating that the two ring systems are not coplanar. The crystal structure is stabilized by intermolecular O—H···O and N—H···O hydrogen-bonding interactions (Fig. 2, Table. 1).

### Experimental

To a ethanol solution (30 ml) of 3-aminobenzoic acid (3.00 mmol) added 3.00 mmol 2-formylbenzoic acid. The mixture solution was stirred at 343 K for 2.5 h. Then, sodium ethoxide (6.6 mmol) was added to the reactor and stirring for 0.5 h. Bis(tributyltin)oxide (0.3 mmol) was then added to the reactor and the reaction mixture was stirred for 6 h. The resulting clear solution was evaporated under vacuum. The product was crystallized from a solution of dichloromethane/methanol (1:1) yielding the title compound unexpectedly. Anal. Calcd (%) for C_15_H_11_N_1_O_4_ (Mr = 269.25): C, 66.91; H, 4.12; N, 5.20; O, 23.77. Found (%): C, 66.87; H, 4.13; N, 5.21; O, 23.79.

### Refinement

All the H atoms were positioned geometrically and constrained to ride on their parent atoms, with C—H = 0.93 (Ar—H), 0.86 (N—H) and 0.82 (O—H) Å.

### Crystal data

**Table:**

C_15_H_11_NO_4_	*F*(000) = 560
*M**_r_* = 269.25	*D*_x_ = 1.449 Mg m^−^^3^
Monoclinic, *P*2_1_/*n*	Mo *K*α radiation, λ = 0.71073 Å
Hall symbol: -P 2yn	Cell parameters from 1143 reflections
*a* = 10.9025 (15) Å	θ = 2.7–21.4°
*b* = 8.1595 (12) Å	µ = 0.11 mm^−^^1^
*c* = 14.2654 (18) Å	*T* = 298 K
β = 103.463 (1)°	Block, colorless
*V* = 1234.2 (3) Å^3^	0.27 × 0.19 × 0.17 mm
*Z* = 4	

### Data collection

**Table:**

Siemens SMART CCD area-detector diffractometer	2171 independent reflections
Radiation source: fine-focus sealed tube	1206 reflections with *I* > 2σ(*I*)
graphite	*R*_int_ = 0.038
φ and ω scans	θ_max_ = 25.0°, θ_min_ = 2.1°
Absorption correction: multi-scan (*SADABS*; Sheldrick, 1996)	*h* = −9→12
*T*_min_ = 0.972, *T*_max_ = 0.982	*k* = −9→9
6011 measured reflections	*l* = −16→14

### Refinement

**Table:**

Refinement on *F*^2^	Primary atom site location: structure-invariant direct methods
Least-squares matrix: full	Secondary atom site location: difference Fourier map
*R*[*F*^2^ > 2σ(*F*^2^)] = 0.041	Hydrogen site location: inferred from neighbouring sites
*wR*(*F*^2^) = 0.107	H-atom parameters constrained
*S* = 0.90	*w* = 1/[σ^2^(*F*_o_^2^) + (0.0513*P*)^2^] where *P* = (*F*_o_^2^ + 2*F*_c_^2^)/3
2171 reflections	(Δ/σ)_max_ < 0.001
181 parameters	Δρ_max_ = 0.23 e Å^−^^3^
0 restraints	Δρ_min_ = −0.13 e Å^−^^3^

### Fractional atomic coordinates and isotropic or equivalent isotropic displacement parameters (Å^2^)

**Table:**

	*x*	*y*	*z*	*U*_iso_*/*U*_eq_	
N1	0.77236 (15)	0.2945 (2)	0.94157 (13)	0.0511 (5)	
H1	0.7596	0.2454	0.8868	0.061*	
O1	0.99795 (14)	0.5570 (2)	1.25378 (11)	0.0745 (6)	
H1A	1.0238	0.6014	1.3062	0.112*	
O2	1.19133 (13)	0.45300 (19)	1.28827 (10)	0.0592 (5)	
O3	0.62455 (14)	0.2734 (2)	1.04292 (10)	0.0614 (5)	
O4	0.44272 (16)	0.1671 (2)	1.06184 (12)	0.0788 (6)	
C1	1.0886 (2)	0.4649 (3)	1.23355 (15)	0.0469 (6)	
C2	1.05166 (19)	0.3785 (3)	1.14008 (14)	0.0407 (5)	
C3	0.92786 (18)	0.3817 (3)	1.08532 (15)	0.0425 (5)	
H3	0.8673	0.4427	1.1063	0.051*	
C4	0.89469 (18)	0.2949 (3)	1.00014 (15)	0.0402 (6)	
C5	0.9866 (2)	0.2051 (3)	0.96999 (16)	0.0494 (6)	
H5	0.9656	0.1478	0.9121	0.059*	
C6	1.1080 (2)	0.2000 (3)	1.02457 (17)	0.0522 (6)	
H6	1.1682	0.1378	1.0039	0.063*	
C7	1.1418 (2)	0.2860 (3)	1.10972 (17)	0.0491 (6)	
H7	1.2243	0.2821	1.1465	0.059*	
C8	0.5015 (2)	0.2389 (3)	1.01183 (17)	0.0555 (7)	
C9	0.67070 (19)	0.3691 (3)	0.96714 (15)	0.0478 (6)	
H9	0.6952	0.4794	0.9916	0.057*	
C10	0.55418 (18)	0.3801 (3)	0.88699 (15)	0.0422 (6)	
C11	0.45572 (18)	0.3033 (3)	0.91465 (15)	0.0436 (6)	
C12	0.3365 (2)	0.2979 (3)	0.85393 (16)	0.0558 (7)	
H12	0.2700	0.2468	0.8729	0.067*	
C13	0.3193 (2)	0.3700 (3)	0.76518 (16)	0.0592 (7)	
H13	0.2400	0.3681	0.7232	0.071*	
C14	0.4181 (2)	0.4451 (3)	0.73746 (16)	0.0590 (7)	
H14	0.4042	0.4926	0.6766	0.071*	
C15	0.53674 (19)	0.4520 (3)	0.79711 (16)	0.0514 (6)	
H15	0.6029	0.5032	0.7777	0.062*	

### Atomic displacement parameters (Å^2^)

**Table:**

	*U*^11^	*U*^22^	*U*^33^	*U*^12^	*U*^13^	*U*^23^
N1	0.0355 (11)	0.0706 (14)	0.0439 (11)	0.0046 (9)	0.0027 (8)	−0.0129 (10)
O1	0.0506 (10)	0.1082 (15)	0.0594 (11)	0.0126 (10)	0.0017 (8)	−0.0300 (10)
O2	0.0406 (9)	0.0749 (12)	0.0529 (10)	−0.0040 (8)	−0.0077 (7)	0.0057 (9)
O3	0.0450 (10)	0.0930 (13)	0.0429 (10)	0.0050 (9)	0.0033 (7)	0.0062 (9)
O4	0.0656 (12)	0.1171 (17)	0.0559 (11)	−0.0037 (10)	0.0183 (9)	0.0242 (11)
C1	0.0398 (13)	0.0544 (15)	0.0446 (14)	−0.0052 (12)	0.0062 (11)	0.0065 (12)
C2	0.0365 (12)	0.0426 (13)	0.0410 (13)	−0.0027 (10)	0.0050 (10)	0.0029 (11)
C3	0.0359 (12)	0.0450 (14)	0.0450 (13)	0.0024 (10)	0.0065 (10)	−0.0004 (11)
C4	0.0333 (12)	0.0438 (14)	0.0412 (13)	−0.0018 (10)	0.0043 (10)	0.0009 (11)
C5	0.0443 (14)	0.0548 (15)	0.0499 (15)	−0.0004 (12)	0.0125 (11)	−0.0056 (12)
C6	0.0403 (14)	0.0549 (16)	0.0630 (16)	0.0059 (11)	0.0155 (12)	−0.0003 (13)
C7	0.0329 (13)	0.0529 (15)	0.0589 (16)	0.0003 (11)	0.0057 (10)	0.0071 (13)
C8	0.0464 (15)	0.0747 (19)	0.0448 (15)	0.0040 (13)	0.0091 (12)	0.0034 (13)
C9	0.0400 (13)	0.0575 (15)	0.0439 (14)	0.0017 (11)	0.0058 (10)	−0.0028 (12)
C10	0.0331 (12)	0.0499 (14)	0.0421 (13)	0.0039 (10)	0.0054 (10)	−0.0064 (11)
C11	0.0365 (13)	0.0559 (15)	0.0378 (13)	0.0021 (11)	0.0075 (10)	−0.0010 (11)
C12	0.0373 (13)	0.0786 (19)	0.0522 (15)	−0.0055 (12)	0.0120 (11)	−0.0035 (13)
C13	0.0375 (14)	0.0906 (19)	0.0448 (15)	0.0049 (13)	0.0002 (11)	−0.0022 (14)
C14	0.0477 (15)	0.0826 (19)	0.0454 (14)	0.0101 (13)	0.0082 (12)	0.0117 (14)
C15	0.0402 (13)	0.0630 (16)	0.0509 (15)	0.0002 (11)	0.0108 (11)	0.0046 (13)

### Geometric parameters (Å, °)

**Table:**

N1—C9	1.386 (3)	C6—C7	1.377 (3)
N1—C4	1.399 (2)	C6—H6	0.9300
N1—H1	0.8600	C7—H7	0.9300
O1—C1	1.326 (2)	C8—C11	1.458 (3)
O1—H1A	0.8200	C9—C10	1.501 (3)
O2—C1	1.211 (2)	C9—H9	0.9800
O3—C8	1.341 (3)	C10—C11	1.378 (3)
O3—C9	1.512 (3)	C10—C15	1.382 (3)
O4—C8	1.215 (3)	C11—C12	1.384 (3)
C1—C2	1.479 (3)	C12—C13	1.369 (3)
C2—C7	1.386 (3)	C12—H12	0.9300
C2—C3	1.394 (3)	C13—C14	1.375 (3)
C3—C4	1.380 (3)	C13—H13	0.9300
C3—H3	0.9300	C14—C15	1.374 (3)
C4—C5	1.388 (3)	C14—H14	0.9300
C5—C6	1.371 (3)	C15—H15	0.9300
C5—H5	0.9300		
			
C9—N1—C4	123.46 (18)	O4—C8—C11	128.4 (2)
C9—N1—H1	118.3	O3—C8—C11	109.5 (2)
C4—N1—H1	118.3	N1—C9—C10	114.38 (18)
C1—O1—H1A	109.5	N1—C9—O3	112.41 (18)
C8—O3—C9	110.22 (17)	C10—C9—O3	102.25 (17)
O2—C1—O1	122.0 (2)	N1—C9—H9	109.2
O2—C1—C2	124.1 (2)	C10—C9—H9	109.2
O1—C1—C2	113.96 (17)	O3—C9—H9	109.2
C7—C2—C3	120.0 (2)	C11—C10—C15	120.71 (18)
C7—C2—C1	118.49 (19)	C11—C10—C9	109.41 (19)
C3—C2—C1	121.4 (2)	C15—C10—C9	129.9 (2)
C4—C3—C2	120.3 (2)	C10—C11—C12	121.0 (2)
C4—C3—H3	119.8	C10—C11—C8	108.61 (18)
C2—C3—H3	119.8	C12—C11—C8	130.3 (2)
C3—C4—C5	118.93 (18)	C13—C12—C11	118.2 (2)
C3—C4—N1	123.01 (19)	C13—C12—H12	120.9
C5—C4—N1	118.05 (19)	C11—C12—H12	120.9
C6—C5—C4	120.8 (2)	C12—C13—C14	120.6 (2)
C6—C5—H5	119.6	C12—C13—H13	119.7
C4—C5—H5	119.6	C14—C13—H13	119.7
C5—C6—C7	120.7 (2)	C15—C14—C13	121.9 (2)
C5—C6—H6	119.7	C15—C14—H14	119.1
C7—C6—H6	119.7	C13—C14—H14	119.1
C6—C7—C2	119.3 (2)	C14—C15—C10	117.6 (2)
C6—C7—H7	120.3	C14—C15—H15	121.2
C2—C7—H7	120.3	C10—C15—H15	121.2
O4—C8—O3	122.1 (2)		

### Hydrogen-bond geometry (Å, °)

**Table:**

*D*—H···*A*	*D*—H	H···*A*	*D*···*A*	*D*—H···*A*
O1—H1A···O4^i^	0.82	1.91	2.712 (2)	166
N1—H1···O2^ii^	0.86	2.16	2.956 (2)	154

Symmetry codes: (i) −*x*+3/2, *y*+1/2, −*z*+5/2; (ii) *x*−1/2, −*y*+1/2, *z*−1/2.
